# Rheumatic Fever in Brazil: What Color Should It Be?

**DOI:** 10.5935/abc.20190178

**Published:** 2019-09

**Authors:** Chris T. Longenecker

**Affiliations:** 1 University Hospitals Harrington Heart and Vascular Institute, Cleveland, Ohio - USA; 2 Case Western Reserve University School of Medicine, Cleveland, Ohio - USA

**Keywords:** Rheumatic Fever/ecomomics Heart Disease/economics, Cardiovascular Surgical Procedures/mortality, Hospitalization/economics, Antibiotic Prophylaxys/economics, Public Health Policy

Rheumatic fever (RF) and its valvular sequela rheumatic heart disease (RHD) have been a scourge on humanity for ages, and it is only relatively recently that high-income countries have seen rates of RF decline dramatically. Yet, the Global Burden of Disease Study estimates that more than 275,000 deaths due to RHD still occur every year around the world, especially in low and middle-income countries including Brazil.^1^

In this issue of the journal, Figueiredo et al.^2^ have presented ambitious modeling of the disease burden and costs of RF and RHD in Brazil. Using data from the Hospital Information System of Brazil, their central findings are that mortality rates for RF and RHD have increased by 215% and 42.5% respectively from 1998 to 2016. Additionally, the estimated cost for procedures related to RF/RHD diagnosis, interventions such as valve surgery, and hospitalizations for RHD complications like stroke and endocarditis was nearly $27 million USD in 2019.

Although staggering, these figures are - by the authors own admission - likely underestimated. There are several reasons for this. First, the authors acknowledge an inadequate disease reporting and surveillance strategy as one possible source of error in the mortality estimates. In addition, the cost analysis only considered direct costs to the medical system, which does not include the indirect costs to the larger economy from lost productivity. Compared to diseases of the elderly (e.g. heart failure), these indirect lost productivity costs are greater for diseases like RF and RHD that claim the lives of children and young adults with a potential lifetime of productive work ahead of them.

In addition to a full assessment of cost burden, studies are needed to assess the cost-effectiveness of interventions to reduce RF/RHD in Brazil. In general, interventions that improve appropriate diagnosis and treatment of group A strep infections (i.e. primary prevention) and benzathine penicillin for all patients with a history of RF/RHD (i.e. secondary prevention) have been shown to be cost-effective in a variety of contexts, including low- and middle-income countries.^3,4^ Assumptions used in the cost-effectiveness modeling should, however, be tailored to the Brazilian context. Is it possible that some Brazilian innovations could improve the cost-effectiveness of RF/RHD prevention? One example is the use of telemedicine for remote ECG diagnosis and appropriate referrals for acute myocardial infarction in Minas Gerais.^5^ This group has already implemented a telemedicine-based strategy for echocardiographic screening for RHD through the PROVAR initiative.^6^

Figueiredo et al.^2^ have included a thoughtful and thorough discussion of the many other issues facing countries who seek to implement a national RF/RHD control program. These include problems with the global penicillin supply chain and the daunting task of addressing social determinants of RF/RHD such as poverty and overcrowding. Yet, missing from their discussion is mention of the World Health Organization’s resolution on RF/RHD issued in April 2018.^7^ This historic resolution calls on the Member States from endemic regions to take eight specific actions: (1) implement a national RHD control program; (2) improve diagnosis and treatment of group A strep pharyngitis; (3) implement secondary prevention monitoring programs; (4) ensure a consistent supply of benzathine penicillin at no cost to patients; (5) educate professionals and the public about RF/RHD prevention; (6) improve access to tertiary care for severe RHD; (7) address known social determinants of RF/RHD; and (8) develop bilateral, regional, and multilateral collaboration and resource mobilization. The resolution also calls on the WHO Secretariat to launch a coordinated global response to RF/RHD, to provide technical assistance to the Member States, to work with pharmaceutical manufacturers to ensure a secure penicillin supply chain, and to convene stakeholders to advance research priorities in vaccine development, disease pathogenesis, and long-acting penicillin formulation. The WHO resolution represents the cresting of a new wave of enthusiasm for RF/RHD prevention among clinicians, policymakers, and - most importantly - affected persons living with RHD. The moment is now to convince governments to invest in this mission. Technical resources are available through organizations such as RHD Action (http://rhdaction.org/) or Reach (www.rheach.org) to assist the Member States in building comprehensive control programs.

The authors are right to compare RF/RHD to other diseases that have similar disease burdens or costs, in order to shine a spotlight on how much less is invested in RF/RHD prevention compared to other diseases. Breast cancer and prostate cancer were highlighted in this analysis; but one could also consider the disease burden and funding that has been dedicated to global infectious diseases. For example, annual global mortality from malaria is only three times greater than RF/RHD, but research and development funding for malaria is over 500 times greater.^8^ The disparity is even worse for HIV/AIDS.

The voice of the RHD community in Brazil and around the world is getting stronger and demanding the attention of public health experts. Breast cancer has Pink October and prostate cancer has Blue November, so what color should be given to a RF/RHD public health campaign in Brazil? Red - to reflect the urgency and severity of the situation? Perhaps it would get confused with HIV/AIDS. Green - to reflect the tropical areas that are so afflicted by the disease worldwide? But this color lacks the sense of urgency required. What about orange, the universal color of warning signs? Ultimately, it is for Brazil to decide, and the global RF/RHD community will be there to support you when you do.
